# Causes and Timing of Mortality and Morbidity Among Late Presenters Starting Antiretroviral Therapy in the REALITY Trial

**DOI:** 10.1093/cid/cix1141

**Published:** 2018-03-04

**Authors:** Frank A Post, Alexander J Szubert, Andrew J Prendergast, Victoria Johnston, Hermione Lyall, Felicity Fitzgerald, Victor Musiime, Godfrey Musoro, Priscilla Chepkorir, Clara Agutu, Jane Mallewa, Chathurika Rajapakse, Helen Wilkes, James Hakim, Peter Mugyenyi, A Sarah Walker, Diana M Gibb, Sarah L Pett, Reduction of EArly mortaLITY in HIV-infected adults and children starting antiretroviral therapy (REALITY) Trial Team, D Gibb, M Thomason, A S Walker, S Pett, A Szubert, A Griffiths, H Wilkes, C Rajapakse, M Spyer, A Prendergast, N Klein, N Van Looy, E Little, K Fairbrother, F Cowan, J Seeley, S Bernays, R Kawuma, Z Mupambireyi, F Kyomuhendo, S Nakalanzi, J Peshu, S Ndaa, J Chabuka, N Mkandawire, L Matandika, C Kapuya, I Weller, E Malianga, C Mwansambo, F Miiro, P Elyanu, E Bukusi, E Katabira, O Mugurungi, D Gibb, J Hakim, A Etyang, P Mugyenyi, J Mallewa, T Peto, P Musoke, J Matenga, S Phiri, H Lyall, V Johnston, F Fitzgerald, F Post, F Ssali, A Prendergast, A Arenas-Pinto, A Turkova, A Bamford

**Affiliations:** 1 King’s College Hospital NHS Foundation Trust, London; 2 Medical Research Council Clinical Trials Unit at University College London; 3 Queen Mary University of London; 4 London School of Hygiene and Tropical Medicine; 5 Imperial College Healthcare NHS Trust, London; 6 University College London Great Ormond Street Institute of Child Health, United Kingdom; 7 Joint Clinical Research Centre, Kampala, Uganda; 8 University of Zimbabwe, Harare; 9 Moi University School of Medicine, Eldoret; 10 Kenya Medical Research Institute–Wellcome Trust Research Programme, Kilifi; 11 Department of Medicine, College of Medicine and Malawi-Liverpool–Wellcome Trust Clinical Research Programme, Blantyre; 12 Institute for Global Health, University College London, United Kingdom; 13 Kirby Institute for Infection and Immunity in Society, University of New South Wales, Sydney, Australia

**Keywords:** HIV, Africa, antiretroviral therapy, mortality, morbidity

## Abstract

**Background:**

In sub-Saharan Africa, 20%–25% of people starting antiretroviral therapy (ART) have severe immunosuppression; approximately 10% die within 3 months. In the Reduction of EArly mortaLITY (REALITY) randomized trial, a broad enhanced anti-infection prophylaxis bundle reduced mortality vs cotrimoxazole. We investigate the contribution and timing of different causes of mortality/morbidity.

**Methods:**

Participants started ART with a CD4 count <100 cells/µL; enhanced prophylaxis comprised cotrimoxazole plus 12 weeks of isoniazid + fluconazole, single-dose albendazole, and 5 days of azithromycin. A blinded committee adjudicated events and causes of death as (non–mutually exclusively) tuberculosis, cryptococcosis, severe bacterial infection (SBI), other potentially azithromycin-responsive infections, other events, and unknown.

**Results:**

Median pre-ART CD4 count was 37 cells/µL. Among 1805 participants, 225 (12.7%) died by week 48. Fatal/nonfatal events occurred early (median 4 weeks); rates then declined exponentially. One hundred fifty-four deaths had single and 71 had multiple causes, including tuberculosis in 4.5% participants, cryptococcosis in 1.1%, SBI in 1.9%, other potentially azithromycin-responsive infections in 1.3%, other events in 3.6%, and unknown in 5.0%. Enhanced prophylaxis reduced deaths from cryptococcosis and unknown causes (*P* < .05) but not tuberculosis, SBI, potentially azithromycin-responsive infections, or other causes (*P* > .3); and reduced nonfatal/fatal tuberculosis and cryptococcosis (*P* < .05), but not SBI, other potentially azithromycin-responsive infections, or other events (*P* > .2).

**Conclusions:**

Enhanced prophylaxis reduced mortality from cryptococcosis and unknown causes and nonfatal tuberculosis and cryptococcosis. High early incidence of fatal/nonfatal events highlights the need for starting enhanced-prophylaxis with ART in advanced disease.

**Clinical Trials Registration:**

ISRCTN43622374.

In sub-Saharan Africa, 20%–25% of human immunodeficiency virus (HIV)–infected individuals starting antiretroviral therapy (ART) have a CD4 count <100 cells/µL [1]; approximately 10% die within 3 months [2–5]. In the recently reported Reduction of EArly mortaLITY in HIV-infected adults and children starting ART (REALITY) randomized trial, a broad enhanced anti-infection prophylaxis bundle reduced all-cause mortality vs cotrimoxazole alone in adults and children aged ≥5 years initiating ART with a CD4 count <100 cells/µL [6]. Here, we investigate in detail the relative contribution of different causes of mortality and morbidity. In particular, we investigate the timing of different causes of mortality/morbidity, as most studies of early mortality have analyzed causes of death over fixed time scales after starting ART [5] and not reported changes in cause-specific mortality rates over time after starting ART.

## METHODS

The REALITY trial (ISRCTN43622374) recruited 1805 HIV-infected ART-naive adults and children ≥5 years (98% aged ≥13 years) from Zimbabwe, Uganda, Malawi, and Kenya with a CD4 count <100 cells/µL. In brief, participants were randomized 1:1 to enhanced prophylaxis vs standard prophylaxis (cotrimoxazole) [6], together with World Health Organization (WHO)–recommended combination ART comprising 2 nucleoside reverse transcriptase inhibitors (NRTIs) (tenofovir disoproxil fumarate + emtricitabine or zidovudine + lamivudine in adults) and 1 nonnucleoside reverse transcriptase inhibitor (NNRTI) (predominantly efavirenz). Enhanced prophylaxis comprised single-dose albendazole, 5 days of azithromycin, 12 weeks of fluconazole (100 mg), and 12 weeks of fixed-dose combination of cotrimoxazole (800/160 mg)/isoniazid (300 mg)/pyridoxine (25 mg) once daily. For children aged 5–12 years, doses were halved (except albendazole). Isoniazid/pyridoxine use post–week 12 depended on national isoniazid preventive therapy guidelines. Participants already receiving or needing antimicrobial treatment or prophylaxis received it outside the randomized design, and received other prophylaxis according to randomization. Participants were factorially randomized 1:1 to 12 weeks of raltegravir vs no raltegravir, and 12 weeks of ready-to-use supplementary food vs standard nutritional support; neither affected mortality (previously reported in [7, 8]). Adults and children’s guardians gave written informed consent; older children gave additional assent following national guidelines. REALITY was approved by ethics committees in Zimbabwe, Uganda, Malawi, Kenya, and the United Kingdom.

Clinical events occurring through 48 weeks were ascertained at trial visits (see Supplementary Methods). Adverse events (AEs) were graded following standard tables [9, 10]. Serious adverse events (SAEs) were those leading to death, being life-threatening, causing/prolonging hospitalization, or causing permanent disability, or were other medical conditions with a risk of 1 of these categories.

### Endpoint Review

An endpoint review committee (ERC) (majority independent members) adjudicated causes of death and nonfatal events (grade 3/4 AEs, SAEs, WHO stage 3/4 events) using clinical narratives written by the treating physician and reviewed by the center’s principal investigator. Events were adjudicated against protocol-defined criteria and grading tables [9, 10] and blinded to interventional drugs received (prophylaxis, raltegravir, and supplementary food). Events were also assessed for compatibility with immune reconstitution inflammatory syndrome (IRIS). Events were considered IRIS compatible if there was atypical or exaggerated presentation of an opportunistic infection or tumor soon after ART initiation. Viral loads (VL) were only measured retrospectively on stored plasma, so were not available contemporaneously; the earliest CD4 count was done at week 4 and thus was not available for events before this. As access to diagnostic testing was limited, previously published definitions [11, 12] were used but in modified form; the ERC relied heavily on the description of the clinical presentation and any radiology/microbiology/histology provided by site, and the timing of the presentation and its evolution in relation to ART initiation. An unblinded clinical reviewer at the coordinating center reviewed grade 3/4 laboratory (nonclinical) events.

### Statistical Analysis

The ERC classified causes of death and events, blinded to randomized group, as related to tuberculosis, cryptococcosis, severe bacterial infection (SBI; including pneumonia/meningitis), other infections potentially responsive to azithromycin (denoted “potentially azithromycin-responsive infections”: diarrhea, malaria, encephalitis, cerebral toxoplasmosis, or other indeterminate severe cerebral events, intracerebral lesions, septic abortion), other events (either noninfectious or infections not responsive to azithromycin), and unknown (deaths only). Indeterminate severe cerebral events and intracerebral lesions were considered azithromycin responsive as these could potentially be toxoplasmosis. Categories were not mutually exclusive.

Analyses were conducted overall and, as enhanced prophylaxis reduced mortality, considering effects of enhanced prophylaxis vs cotrimoxazole alone. Analyses of causes of death, and first fatal/nonfatal events (including grade 3/4 AE, SAE, and WHO stage 3/4 events) used competing risks methods [13]. All follow-up from randomization to the earliest of 48 weeks, or last contact with clinic staff, was included. To estimate continuously varying cause-specific rates, we used flexible parametric models (see details in Supplementary Methods). Event rates were estimated to rise for at least 7 days after randomization, plausibly reflecting exclusion of patients without capacity to consent due to severe comorbidities, leading to few deaths being observed during the first few days after randomization. Therefore, while analysis included all follow-up, interpretation of results focused on follow-up post–7 days (Supplementary Methods). Finally, as enhanced prophylaxis significantly reduced death from cryptococcosis and from unknown causes [6], predictors of these deaths were identified using competing risks methods [13] (details in Supplementary Methods).

All analyses were performed using Stata 15.1 software (StataCorp, College Station, Texas). *P* values are 2-sided.

## RESULTS

A total of 1805 participants initiated ART between June 2013 and April 2015. Median age was 36 years, median CD4 count was 37 cells/µL, and 1326 of 1792 (74.0%) had HIV VL ≥100000 copies/mL (Table 1). Despite profound immunosuppression, 854 (47.3%) had only WHO stage 1/2 disease [6].

**Table 1.** Characteristics at Enrollment

Factor		Standard Prophylaxis (n = 899)	Enhanced Prophylaxis (n = 906)	All (N = 1805)
Country	Kenya	174 (19.4)	177 (19.5)	351 (19.4)
	Malawi	128 (14.2)	127 (14.0)	255 (14.1)
	Uganda	313 (34.8)	317 (35.0)	630 (34.9)
	Zimbabwe	284 (31.6)	285 (31.5)	569 (31.5)
Male sex		484 (53.8)	477 (52.6)	961 (53.2)
Age, y		36 (30–42) [range, 5–78]	36 (29–42) [range, 6–71]	36 (29–42) [range, 5–78]
Age 5–17 y		33 (3.7)	39 (4.3)	72 (4.0)
WHO stage	1	153 (17.0)	147 (16.2)	300 (16.6)
	2	265 (29.5)	289 (31.9)	554 (30.7)
	3	349 (38.8)	342 (37.7)	691 (38.3)
	4	132 (14.7)	128 (14.1)	260 (14.4)
CD4 count^a^, cells/µL		36 (16–60)	38 (16–64)	37 (16–63)
CD8 count, cells/µL (n = 1693)		473 (302–734)	497 (317–721)	482 (308–726)
HIV viral load, copies/mL (n = 1792)		250000 (92460–544240)	246130 (97620–654160)	249770 (95540–604960)
HIV viral load ≥100000 copies/mL		655/893 (73.3)	671/899 (74.6)	1326/1792 (74.0)
BMI, kg/m^2^ (n = 1797)		19.3 (17.4–21.5)	19.1 (17.1–21.3)	19.2 (17.2–21.4)
Hemoglobin, g/dL (n = 1800)		11.2 (9.6–12.7)	11.1 (9.5–12.8)	11.2 (9.6–12.7)
Creatinine clearance^b^, mL/min (n = 1793)		97.1 (77.8–120.1)	97.3 (76.6–121.9)	97.3 (77.3–121.1)
Albumin, g/L (n = 1751)		35 (30–40)	35 (30–40)	35 (30–40)
Bilirubin, µmol/L (n = 1768)		6.0 (3.9–8.4)	6.0 (3.8–9.0)	6.0 (3.8–8.9)
WBC count, 10^9^/L (n = 1800)		3.6 (2.7–4.7)	3.5 (2.7–4.7)	3.5 (2.7–4.7)
Lymphocyte count, 10^9^/L (n = 1790)		0.9 (0.7–1.3)	1.0 (0.7–1.4)	1.0 (0.7–1.4)
Neutrophil count, 10^9^/L (n = 1783)		1.8 (1.2–2.6)	1.7 (1.2–2.5)	1.7 (1.2–2.6)
Platelet count, 10^9^/L (n = 1789)		252 (184–333)	252 (183–352)	252 (183–342)
Water source (n = 1761)				
Protected well		327 (37.3)	296 (33.5)	623 (35.4)
Domestic tap: private standpipe		246 (28.1)	258 (29.2)	504 (28.6)
Domestic tap: communal standpipe		98 (11.2)	89 (10.1)	187 (10.6)
Communal tap		119 (13.6)	136 (15.4)	255 (14.5)
Lake/river/stream		32 (3.6)	41 (4.6)	73 (4.1)
Unprotected well		55 (6.3)	64 (7.2)	119 (6.8)
Household toilet type (n = 1794)				
Pit/latrine		635 (70.6)	616 (68.8)	1251 (69.7)
Flush		264 (29.4)	279 (31.2)	543 (30.3)

Data are presented as No. (%) or median (interquartile range).

Abbreviations: BMI, body mass index; HIV, human immunodeficiency virus; WBC, white blood cell; WHO, World Health Organization.

^a^Mean of screening and enrollment values. Eligibility required screening CD4 count to be <100 cells/µL, so baseline could be ≥100 cells/µL depending on the CD4 at enrollment.

^b^Estimated using Cockcroft-Gault, normalized to 1.73 m^2^.

### Deaths

**Figure 1.**
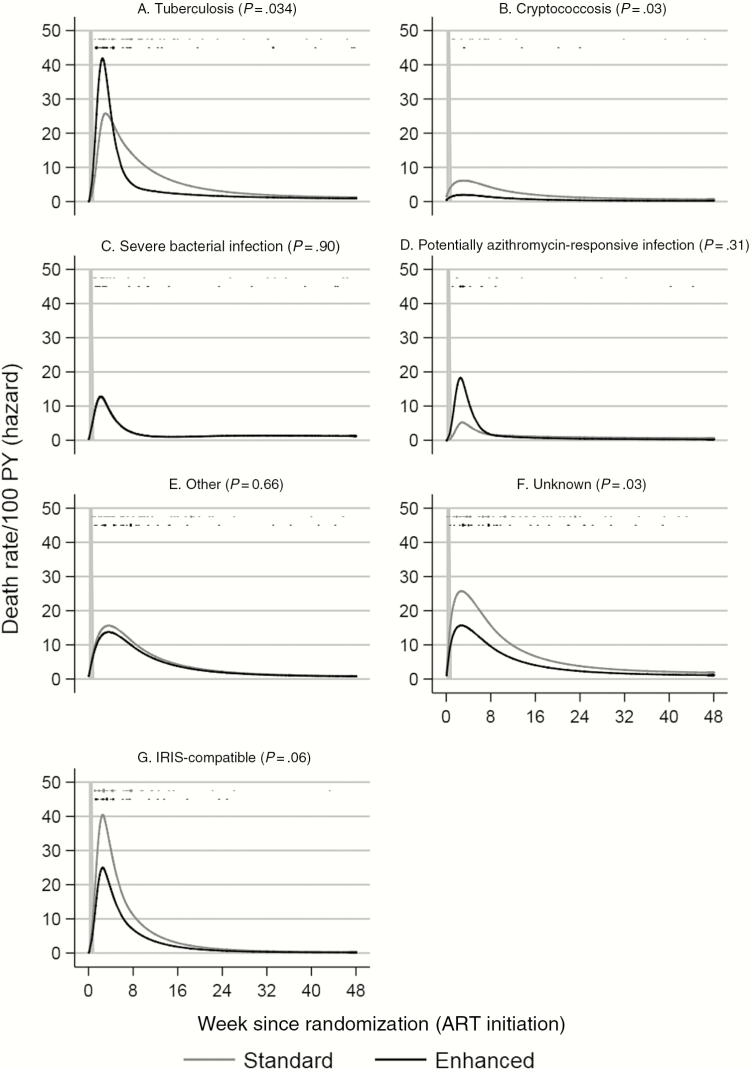
Rates (per 100 person-years [PY]) of fatal events over time from antiretroviral therapy (ART) initiation due to tuberculosis (*A*), cryptococcosis (*B*), severe bacterial infections (*C*), potentially azithromycin-responsive infections (*D*), other events (*E*), unknown deaths (*F*), and immune reconstitution inflammatory syndrome (IRIS)–compatible deaths (*G*). Vertical dashes show times when events occurred; length proportional to number of events (≥10 shown as 10). Order as legend (standard, enhanced). All-cause mortality shown in Supplementary Figure 4*A* of [6].

Of the 225 deaths by 48 weeks on ART, 154 (68.4%) were assigned to a single category and 71 (31.6%) to multiple categories. Overall, the most common cause of death (non–mutually exclusive) was unknown (n = 88 participants [5.0% cumulative incidence]; many died at home), then tuberculosis (n = 80 [4.5%]), other events (n = 63 [3.6%]), SBI (n = 33 [1.9%]), other potentially azithromycin-responsive infections (n = 23 [1.3%]), and cryptococcosis (n = 20 [1.1%]). Sixty-seven (29.8%) deaths were adjudicated as compatible with IRIS. Enhanced prophylaxis reduced all-cause mortality by 24 weeks by 27% (hazard ratio, 0.73; 95% confidence interval, .55–.98) log-rank *P* = .03), which was maintained through 48 weeks (see Figure 2*B* in [6]). Overall, enhanced prophylaxis reduced deaths from unknown causes (3.8% vs 6.1% standard prophylaxis, competing risks *P* = .03) and from cryptococcosis (0.6% vs 1.8%, respectively; *P* = .03); with a similar trend in IRIS-compatible deaths (2.9% vs 4.6%, respectively; *P* = .06) (Supplementary Figure 1). There was no evidence that enhanced prophylaxis affected deaths from tuberculosis (*P* = .34), SBI (*P* = .90), potentially azithromycin-responsive infections (*P* = .31), and other events (*P* = .66). However, regardless of prophylaxis received, mortality rates were highest during the first 4 weeks on ART, dropping substantially through week 8, before dropping further through weeks 24 and 48 (Figure 1; Supplementary Table 1).

### Fatal and Nonfatal Events

**Figure 2.**
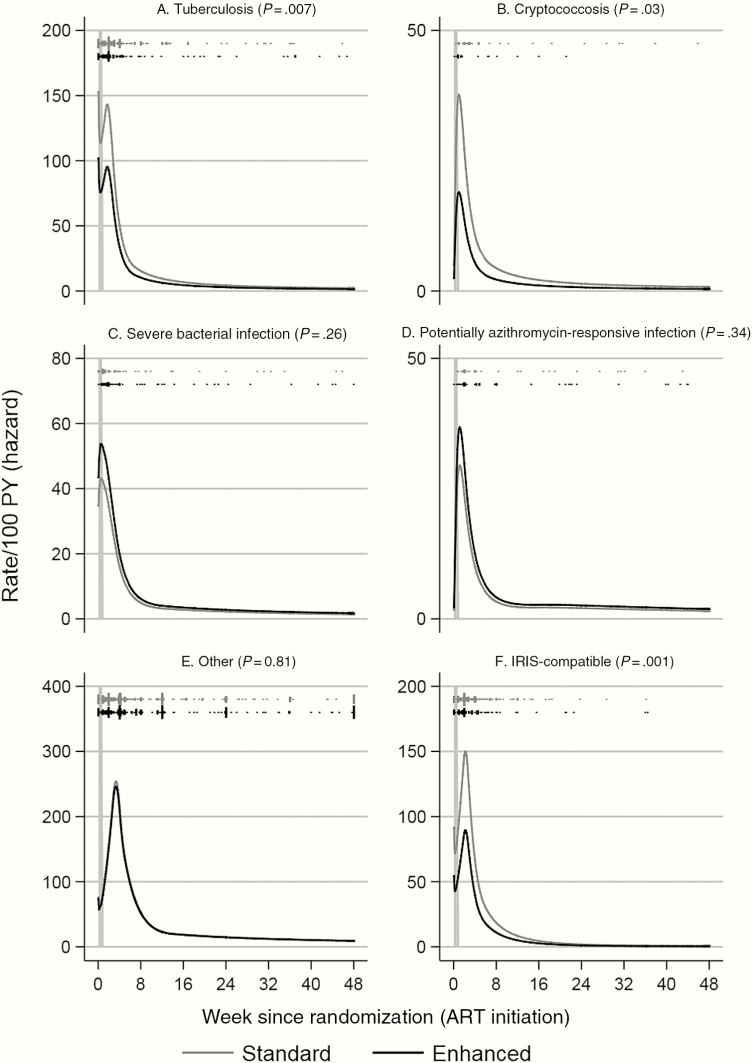
Rates (per 100 person-years [PY]) of combined fatal plus nonfatal events over time from antiretroviral therapy (ART) initiation: tuberculosis (*A*), cryptococcosis (*B*), severe bacterial infections (*C*), potentially azithromycin-responsive infections (*D*), other events (*E*), and immune reconstitution inflammatory syndrome (IRIS)–compatible events (*F*). Vertical dashes show times when events occurred; length proportional to number of events (≥10 shown as 10). Order as legend (standard, enhanced).

By 48 weeks on ART, 963 first fatal or nonfatal (grade 3/4 AE, SAE, WHO stage 3/4) events of each category occurred in 674 (37.3%) participants. The most common were other events (n = 479 participants [27.0%]) (Supplementary Table 2), followed by tuberculosis (n = 195 [10.9%]), SBIs (n = 91 [5.1%]), other potentially azithromycin-responsive infections (n = 65 [3.6%]), and cryptococcosis (n = 44 [2.5%]). One hundred seventy-five (9.8%) participants had IRIS-compatible events. Enhanced prophylaxis reduced tuberculosis (8.9% vs 13.0% standard prophylaxis; *P* = .007), cryptococcosis (1.7% vs 3.3%, respectively; *P* = .03), and IRIS-compatible events (7.5% vs 12.2%, respectively; *P* = .001) (Supplementary Figure 2), but not SBIs (*P* = .26), potentially azithromycin-responsive infections (*P* = .34), or other events (*P* = .81). Although occurring at much higher rates (Figure 2), similarly to deaths, first fatal or nonfatal events predominantly occurred very shortly after ART initiation (median, 4.0 weeks; interquartile range [IQR], 2.0–11.7 weeks), even for the large group of other events which included probable noninfectious events and those not traditionally considered HIV-related (Supplementary Table 2). Rates for all types of events declined exponentially from the third week on ART.

At 48 weeks post–ART initiation, rates of fatal/nonfatal events were lowest (<1/100 person-years [PY] for cryptococcosis; Supplementary Table 1). Rates were moderate (1–5/100 PY) for tuberculosis, SBIs, and other potentially azithromycin-responsive infections. Rates were highest (>5/100 PY) for other events. Death rates were highest (1–5/100 PY) for deaths from tuberculosis, SBIs, and unknown causes.

### Predictors of Deaths From Cryptococcosis and Unknown Causes

Given the impact of enhanced prophylaxis on deaths from cryptococcosis and unknown causes, we considered whether predictors of these 2 categories of death were similar, suggesting unascertained cryptococcal disease could be driving these effects. Deaths from cryptococcosis were more common in standard prophylaxis (*P* = .03) and participants with lower baseline CD4 count (*P* = .02) or self-reported vomiting (*P* = .005) (Supplementary Table 3). Deaths from unknown causes were more common in participants administered standard prophylaxis (*P* = .03); those with lower baseline CD4 count (*P* = .05), creatinine clearance (*P* < .001), or albumin (*P* = .01); higher bilirubin (*P* = .05); without previous healthcare contact (*P* = .04); or with wasting/severe weight loss (*P* = .02), participant-reported fever (*P* = .08), or problems with mobility (*P* = .05) or self-care (*P* = .05). There was no effect of water source or household toilet type on either cause of death (*P* > .3).

#### Last Postbaseline VL and CD4 Count Before Fatal and Nonfatal Events of Each Cause

Considering the 1716 participants alive and in follow-up at week 4, median last postbaseline VL before death was 95 (IQR, <50–522) copies/mL (Supplementary Table 4), with relatively little variation between causes, suggesting excellent VL response, given high values pre-ART. However, median CD4 count was only 59 (IQR, 32–101) cells/µL, also with little variation between causes, demonstrating that most remained severely immunocompromised. Results were similar for nonfatal events (Supplementary Table 4).

## DISCUSSION

In the REALITY trial of patients starting ART with severe immunosuppression in sub-Saharan Africa, morbidity/mortality rates were high in the first month after starting ART, regardless of type of event, and dropped exponentially thereafter. However, even within a randomized trial with intensive follow-up, structured clinical narratives, and independent endpoint review, causes of death were difficult to ascertain and the commonest category was unknown. Furthermore, multiple causes were identified in almost one-third of deaths, highlighting the complexity of clinical presentations in patients starting ART with advanced immunosuppression.

REALITY showed that enhanced prophylaxis reduces mortality by 27% [6], due to reductions in deaths from cryptococcosis and unknown causes, but not from tuberculosis or severe, but mainly presumptive, bacterial infections. Nonfatal tuberculosis events were, however, significantly reduced, leaving only azithromycin and albendazole, of the 5 different antimicrobial agents, without clear benefit. We therefore explored whether deaths due to infections potentially responsive to azithromycin (predominantly toxoplasmosis, diarrhea/gastroenteritis, and malaria) were reduced by enhanced prophylaxis, but found no evidence of this. The apparent lack of impact on bacterial infections raises questions about using 5 days of azithromycin within enhanced prophylaxis [14], particularly given concerns about antimicrobial resistance. However, it is not possible to exclude azithromycin on the basis of these findings alone. For example, 29.5% of deaths from unknown causes occurred within the first 4 weeks after ART initiation and many occurred at home, making it difficult to exclude the possibility that azithromycin had a role in reducing undiagnosed SBIs causing these early deaths. Alternatively, azithromycin may have been ineffective against the most common causes of bacterial infections in this population, for example, due to already high rates of antimicrobial resistance [15]. To explore whether deaths from unknown causes could have been caused by cryptococcosis, we identified predictors of mortality from these causes. Although both deaths from cryptococcosis and unknown causes were predicted by low CD4 count, other predictors differed, in particular symptoms (fever) and metabolic derangements, consistent with deaths from unknown causes not being primarily due to undiagnosed cryptococcosis, and at least some being due to SBIs and/or other undiagnosed infections such as tuberculosis and atypical mycobacteria. Finally, the severity of immunodeficiency, timing of highest incidence of fatal and nonfatal events, and observed virological responses to ART raise the possibility that a substantial number of events may have been caused or exacerbated by IRIS. We found significant reductions in IRIS-compatible events overall with enhanced prophylaxis. A key limitation is that we were unable to use published definitions of IRIS [11, 12], and some IRIS events may have been misclassified, due to paucity of clinical, radiological, and/or microbiological supporting information (reflecting available services at the centers). Moreover, VL was only measured retrospectively, so not available contemporaneously; the earliest CD4 was week 4 and not available for events before this. However, the ERC made all adjudications blinded to trial drugs, minimizing the potential for bias in comparisons of randomized groups. Our rates of IRIS were lower than some previous studies [16], particularly given the substantial immunosuppression at ART initiation, but likely included the majority of clinically important events, with missed events likely predominantly being less severe. Antimicrobials may modulate the inflammatory milieu through reductions in subclinical infections, enteropathy, and microbial translocation, or through direct immunomodulatory effects (especially azithromycin), and it is well recognized that inflammation can drive mortality independent of immunosuppression [17–19]. Over the longer term, morbidity became more dominated by noninfectious events with lower fatality; however, these were also common early after ART, highlighting the potential role of inflammation in noninfectious disease processes. Planned analysis of cryptococcal antigen, bacterial DNA, and inflammatory markers using pre-ART plasma samples will test these hypotheses and help inform the optimal choice of antimicrobials for future use.

Of note, 62.7% of participants had no serious morbidity/mortality during their first 48 weeks on ART, despite very low pre-ART CD4 and high VL. Nevertheless, their low CD4 put them at substantially increased risk of impaired short- and long-term immunological response, reinforcing the importance of undertaking baseline CD4 to identify these patients. Where CD4 is becoming less available, our data reinforce the need to further develop CD4 point-of-care tests which at least qualitatively identify the most immunosuppressed individuals. Alternatively, total lymphocyte counts could be used to identify at least some of those at greatest risk [20, 21].

Given current WHO guidance focusing on diagnostic tools to rule out opportunistic infections before ART initiation [14], our pragmatic approach of clinically ruling out infections, followed by an immediate prophylaxis bundle at ART initiation, may have substantial advantages for patients, staff, and programs. It can be implemented without diagnostic tests being available (especially at lower-level health centers) and more reliably ensure that patients start ART on time and have rapid prophylactic cover against the main infections. However, even the enhanced prophylaxis group experienced considerable early mortality. The most common cause of death was unknown; many occurred at home. This emphasizes the need to maintain close contact with immunosuppressed patients starting ART (even if clinically asymptomatic), ensuring they seek urgent medical care if they develop new symptoms. New tools to identify those at greatest risk of death [22], and clinical trials to evaluate other short-term anti-inflammatory interventions in addition to ART, are also needed.

## Supplementary Data

Supplementary materials are available at *Clinical Infectious Diseases* online. Consisting of data provided by the authors to benefit the reader, the posted materials are not copyedited and are the sole responsibility of the authors, so questions or comments should be addressed to the corresponding author.

Supplementary Material 1

## Notes

***Acknowledgments.*** We thank all the participants and staff from all the centers participating in the REALITY trial.

***REALITY Trial Group.*** *Participating centers:* Joint Clinical Research Centre (JCRC), Kampala, Uganda (coordinating centre for Uganda): P Mugyenyi, C Kityo, V Musiime, P Wavamunno, E Nambi, P Ocitti, M Ndigendawani. JCRC, Fort Portal, Uganda: S Kabahenda, M Kemigisa, J Acen, D Olebo, G Mpamize, A Amone, D Okweny, A Mbonye, F Nambaziira, A Rweyora, M Kangah and V Kabaswahili. JCRC, Gulu, Uganda: J Abach, G Abongomera, J Omongin, I Aciro, A Philliam, B Arach, E Ocung, G Amone, P Miles, C Adong, C Tumsuiime, P Kidega, B Otto, F Apio. JCRC, Mbale, Uganda: K Baleeta, A Mukuye, M Abwola, F Ssennono, D Baliruno, S Tuhirwe, R Namisi, F Kigongo, D Kikyonkyo, F Mushahara, D Okweny, J Tusiime, A Musiime, A Nankya, D Atwongyeire, S Sirikye, S Mula, N Noowe. JCRC, Mbarara, Uganda: A Lugemwa, M Kasozi, S Mwebe, L Atwine, T Senkindu, T Natuhurira, C Katemba, E Ninsiima, M Acaku J Kyomuhangi, R Ankunda, D Tukwasibwe, L Ayesiga. University of Zimbabwe Clinical Research Centre, Harare, Zimbabwe: J Hakim, K Nathoo, M Bwakura-Dangarembizi, A Reid, E Chidziva, T Mhute, GC Tinago, J Bhiri, S Mudzingwa, M Phiri, J Steamer, R Nhema, C Warambwa, G Musoro, S Mutsai, B Nemasango, C Moyo, S Chitongo, K Rashirai, S Vhembo, B Mlambo, S Nkomani, B Ndemera, M Willard, C Berejena, Y Musodza, P Matiza, B Mudenge, V Guti. KEMRI Wellcome Trust Research Programme, Kilifi, Kenya: A Etyang, C Agutu, J Berkley, K Maitland, P Njuguna, S Mwaringa, T Etyang, K Awuondo, S Wale, J Shangala, J Kithunga, S Mwarumba, S Said Maitha, R Mutai, M Lozi Lewa, G Mwambingu, A Mwanzu, C Kalama, H Latham, J Shikuku, A Fondo, A Njogu, C Khadenge, B Mwakisha. Moi University Clinical Research Centre, Eldoret, Kenya: A Siika, K Wools-Kaloustian, W Nyandiko, P Cheruiyot, A Sudoi, S Wachira, B Meli, M Karoney, A Nzioka, M Tanui, M Mokaya, W Ekiru, C Mboya, D Mwimali, C Mengich, J Choge, W Injera, K Njenga, S Cherutich, M Anyango Orido,G Omondi Lwande, P Rutto, A Mudogo, I Kutto, A Shali, L Jaika, H Jerotich, M Pierre. Department of Medicine and Malawi-Liverpool-Wellcome Trust Clinical Research Programme, College of Medicine, Blantyre, Malawi: J Mallewa, S Kaunda, J Van Oosterhout, B O’Hare, R Heydermann, C Gonzalez, N Dzabala, C Kelly, B Denis, G Selemani, L Nyondo Mipando, E Chirwa, P Banda, L Mvula, H Msuku, M Ziwoya, Y Manda, S Nicholas, C Masesa, T Mwalukomo, L Makhaza, I Sheha, J Bwanali, M Limbuni.

*Trial Coordination and Oversight:* MRC Clinical Trials Unit at UCL, London, UK: D Gibb, M Thomason, A S Walker, S Pett, A Szubert, A Griffiths, H Wilkes, C Rajapakse, M Spyer, A Prendergast, N Klein. Data Management Systems: M Rauchenberger, N Van Looy, E Little, K Fairbrother.

*Social Science Group:* F Cowan, J Seeley, S Bernays, R Kawuma, Z Mupambireyi.

Independent REALITY Trial Monitors: F Kyomuhendo, S Nakalanzi, J Peshu, S Ndaa, J Chabuka, N Mkandawire, L Matandika, C Kapuya.

*Trial Steering Committee:* I Weller (Chair), E Malianga, C Mwansambo, F Miiro, P Elyanu, E Bukusi, E Katabira, O Mugurungi, D Gibb, J Hakim, A Etyang, P Mugyenyi, J Mallewa.

*Data Monitoring Committee:* T Peto (Chair), P Musoke, J Matenga, S Phiri.

*Endpoint Review Committee* (independent members): H Lyall (Co-Chair), V Johnston (Co-Chair), F Fitzgerald, F Post, F Ssali, A Prendergast, A Arenas-Pinto, A Turkova, A Bamford.

***Financial support.*** The REALITY trial was funded by the Joint Global Health Trials Scheme of the UK Department for International Development, the Wellcome Trust and Medical Research Council (MRC) (grant number G1100693). Additional funding support was provided by the PENTA Foundation and core support to the MRC Clinical Trials Unit at University College London (UCL) (grant numbers MC_UU_12023/23 and MC_UU_12023/26). Cipla Ltd, Gilead Sciences, ViiV Healthcare/GlaxoSmithKline, and Merck Sharp & Dohme donated drugs for REALITY, and ready-to-use supplementary food was purchased from Valid International. A. J. P. is funded by the Wellcome Trust (grant number 108065/Z/15/Z). F. F. is supported by a grant from the MRC (grant number MR/K023535/1) and the National Institute for Health Research Biomedical Research Centre at Great Ormond Street Hospital for Children NHS Foundation Trust and UCL. The Malawi-Liverpool–Wellcome Trust Clinical Research Programme, University of Malawi College of Medicine (grant number 101113/Z/13/Z), and Kenya Medical Research Institute (KEMRI)/Wellcome Trust Research Programme, Kilifi (grant number 203077/Z/16/Z) are supported by strategic awards from the Wellcome Trust, United Kingdom. Permission to publish was granted by the Director of KEMRI. This supplement was supported by funds from the Bill & Melinda Gates Foundation.

***Supplement sponsorship.*** This article appears as part of the supplement “Advanced HIV Disease,” sponsored by the World Health Organization.

***Potential conflicts of interest.*** The institution of A. S. W. has received funding from Tibotec and Gilead Sciences for data and safety monitoring board membership and lectures. All other authors report no potential conflicts of interest. All authors have submitted the ICMJE Form for Disclosure of Potential Conflicts of Interest. Conflicts that the editors consider relevant to the content of the manuscript have been disclosed.
