# Supplementary material for: Causes and Timing of Mortality and Morbidity Among Late Presenters Starting Antiretroviral Therapy in the REALITY Trial
Source: Clin Infect Dis. 2018 Mar 4;66(Suppl 2):S132–9. doi: 10.1093/cid/cix1141 (PMC5850430; doi:10.1093/cid/cix1141)
Supplement: Supplementary Material 1 [file cix1141_suppl_supplementary_material_1.docx]

**SUPPLEMENTARY METHODS**

Clinical events occurring through 48 weeks were ascertained at trial visits (nurse visits at weeks 2/4/8/12/18/24/36/48 included a symptom checklist; at weeks 4/12/24/36/48, history and clinical examination by a physician). Participants were also encouraged to return to clinic if unwell. All defaulting participants were traced through home visits and telephone calls. Serious adverse events (SAEs) were those leading to death, being life-threatening, causing/prolonging hospitalization (excluding elective procedures), causing permanent disability, or were other medical conditions with a risk of one of these categories.

**Statistical analysis**

Analyses of causes of death used competing risks methods to estimate the probability of dying from that cause using cumulative incidence functions (analogous to Kaplan-Meier), and the effect of factors on the corresponding (subdistribution) hazard[1]. Incidence of first fatal or non-fatal event of each type was analysed similarly, treating deaths from other causes as competing events and including grade 3/4 AE, SAE, and WHO 3/4 events.

To estimate continuously varying cause-specific death rates, and rates of first fatal/non-fatal events, we used flexible parametric models based on the standard Weibull model[2, 3] which allow event rates to increase then decrease or vice-versa using natural cubic splines. As recommended, the Akaike Information Criterion (AIC) was used to identify the number of interior knots for natural cubic splines (between 0-4)[3]. AIC-based selection of the underlying model was performed adjusted for randomization to enhanced- versus standard-prophylaxis as an explanatory factor since this affected mortality and morbidity[4]. AIC was then used to assess whether the variation in event rates over time differed according to randomized group (Supplementary Table 5). Models for cause-specific death or event rates included all follow-up from randomisation to the earliest of 48 weeks or last contact with clinic staff, and censored participants dying without the event-of-interest at death. Event rates were estimated to rise for at least seven days post-randomization, plausibly reflecting exclusion of patients without capacity to consent due to severe co-morbidities leading to few deaths being observed during the first few days post-randomization. Unpublished simulation studies suggest that such initial rises are a consequence of events typically not being observed in the first few days on ART for such reasons. Therefore, whilst analysis included all follow-up, interpretation of results focused on follow-up post-7-days.

As enhanced-prophylaxis significantly reduced death from cryptococcosis and from unknown causes[4], predictors of these deaths were identified using competing risks methods[1] based on backwards elimination with exit p=0.1 including non-linearity by fractional polynomials where p<0.05 (Stata mfp), forcing randomized group into the model. Factors considered were those in Table 1 or identified as predictors of all-cause mortality in the companion paper in this supplement[5] (haematology/biochemistry truncated at 1st and 99th percentiles). As *Cryptococcus neoformans* is ubiquitous in soil, we also considered available data on water source and household toilet type as potential predictors of deaths from cryptococcosis or unknown causes.

**Supplementary Table 1 Estimated event rates over time from ART initiation (pooling enhanced- and standard-prophylaxis groups)**

|  | **Rate per 100 person-years** | | | |
| --- | --- | --- | --- | --- |
|  | **Week 4** | **Week 8** | **Week 24** | **Week 48** |
| Deaths |  |  |  |  |
| Tuberculosis | 23.8 | 9.4 | 2.3 | 1.1 |
| Cryptococcosis | 4.0 | 2.7 | 0.8 | 0.4 |
| Severe bacterial infection | 8.0 | 2.2 | 1.3 | 1.2 |
| Potentially-azithromycin-responsive infections | 7.6 | 2.0 | 0.7 | 0.4 |
| Other events | 14.6 | 10.1 | 2.1 | 0.8 |
| Unknown causes | 19.6 | 12.6 | 3.0 | 1.5 |
| All | 56.6 | 27.6 | 7.8 | 4.2 |
| All events (fatal and non-fatal) |  |  |  |  |
| Tuberculosis | 42.1 | 12.7 | 3.6 | 1.8 |
| Cryptococcosis | 8.1 | 3.3 | 1.1 | 0.6 |
| Severe bacterial infection | 18.1 | 5.6 | 2.5 | 1.6 |
| Potentially-azithromycin-responsive infections | 11.3 | 3.7 | 2.3 | 1.7 |
| Other events | 212.9 | 52.5 | 14.8 | 9.3 |

**Supplementary Table 2 Other fatal and non-fatal events**

|  | **Number of patients with** | | |
| --- | --- | --- | --- |
| **Event** | **fatal event*** | **non-fatal event**** | **fatal or non-fatal event** |
| Abdominal or epigastric pain | 0 | 1 | 1 |
| Acute abdomen | 1 | 1 | 1 |
| Acute altered conscious level | 0 | 1 | 1 |
| Acute focal neurological event without fever | 0 | 1 | 1 |
| Acute hepatitis | 1 | 14 | 14 |
| Alcohol related event | 1 | 0 | 1 |
| Anaemia with clinical symptoms | 10 | 65 | 67 |
| Anaemia with no clinical symptoms | 0 | 43 | 43 |
| Appendicitis | 0 | 1 | 1 |
| Ascites | 0 | 1 | 1 |
| Benign tumour | 0 | 1 | 1 |
| CMV retinitis | 0 | 1 | 1 |
| Candidiasis of oesophagus, trachea, bronchi or lungs | 3 | 18 | 19 |
| Cardiomyopathy | 1 | 2 | 2 |
| Chest pain | 0 | 1 | 1 |
| Cirrhosis | 1 | 1 | 1 |
| Congestive cardiac failure | 1 | 1 | 1 |
| Cutaneous warts | 0 | 1 | 1 |
| Deep vein thrombosis | 1 | 9 | 9 |
| Dehydration | 0 | 1 | 1 |
| Depression | 0 | 1 | 1 |
| Diabetes - Type II | 2 | 0 | 2 |
| Disorientated/confusion | 0 | 7 | 7 |
| Dizziness | 0 | 2 | 2 |
| Dreams, nightmares | 0 | 1 | 1 |
| Dysphagia, difficulty swallowing | 1 | 2 | 2 |
| Encephalopathy – unspecified | 1 | 1 | 2 |
| Epilepsy, fits, convulsions | 0 | 2 | 2 |
| Gynaecomastia | 0 | 3 | 3 |
| HIV associated nephropathy | 1 | 3 | 3 |
| Haematemesis | 2 | 0 | 2 |
| Headache | 0 | 2 | 2 |
| Hemiparesis | 0 | 2 | 2 |
| Hepatic encephalopathy | 1 | 0 | 1 |
| Hepatic failure - acute | 5 | 5 | 5 |
| Hepatic failure - chronic | 0 | 1 | 1 |
| Hepatitis B | 1 | 4 | 4 |
| Hepatitis cause unknown | 1 | 2 | 3 |
| Herpes Simplex ulceration - skin | 0 | 1 | 1 |
| Human Papillomavirus | 0 | 1 | 1 |
| Hyperkalaemia | 0 | 1 | 1 |
| Hypernatraemia | 0 | 1 | 1 |
| Hypersensitivity reaction | 1 | 9 | 10 |
| Hypertension | 0 | 1 | 1 |
| Hypoglycaemia | 0 | 1 | 1 |
| Hyponatraemia | 1 | 6 | 7 |
| Hypophosphataemia | 0 | 8 | 8 |
| Hypotension/shock/toxic shock | 0 | 1 | 1 |
| Indigestion, oesophageal reflux, gastritis, ulcerative oesophagitis | 0 | 2 | 2 |
| Inter-cranial pressure | 0 | 1 | 1 |
| Intravascular haemolysis | 0 | 1 | 1 |
| Jaundice | 0 | 2 | 2 |
| Kaposi's sarcoma - other | 2 | 5 | 5 |
| Kaposi's sarcoma cutaneous | 2 | 6 | 7 |
| Kaposi's sarcoma lymph nodes | 2 | 4 | 4 |
| Kaposi's sarcoma pulmonary | 0 | 1 | 1 |
| Low albumin | 0 | 1 | 1 |
| Lower urinary tract infection (UTI), cystitis | 1 | 2 | 2 |
| Lymphadenopathy | 0 | 1 | 1 |
| Metabolic disorder - other | 1 | 1 | 1 |
| Myelopathy | 0 | 1 | 1 |
| Nephrotic syndrome | 0 | 1 | 1 |
| Neutropenia | 0 | 127 | 127 |
| Non Hodgkin lymphoma | 1 | 1 | 1 |
| Non-fatal trauma | 0 | 3 | 3 |
| Oral candida | 0 | 10 | 10 |
| Other CNS disease | 0 | 2 | 2 |
| Other solid tumour | 1 | 2 | 2 |
| Overdose (not suicide attempt) | 1 | 1 | 2 |
| PML | 1 | 2 | 2 |
| Pancreatitis | 1 | 1 | 2 |
| Pancytopenia, bone marrow depression | 3 | 5 | 5 |
| Peptic/gastric/duodenal ulcer | 1 | 0 | 1 |
| Peripheral neuropathy - sensory & motor | 0 | 3 | 3 |
| Peripheral neuropathy - sensory only | 0 | 1 | 1 |
| Primary CNS lymphoma | 0 | 1 | 1 |
| Psychosis, mania | 0 | 11 | 11 |
| Pulmonary embolism | 1 | 1 | 2 |
| Pure red cell aplasia | 0 | 1 | 1 |
| Pyelonephritis | 0 | 4 | 4 |
| Raised ALT | 0 | 27 | 27 |
| Raised AST | 0 | 10 | 10 |
| Raised bilirubin | 0 | 1 | 1 |
| Raised creatinine | 0 | 9 | 9 |
| Raised liver enzymes | 0 | 1 | 1 |
| Rash, maculopapular | 0 | 1 | 1 |
| Rash, urticaria | 0 | 1 | 1 |
| Renal failure - acute | 15 | 29 | 30 |
| Renal failure - chronic | 2 | 1 | 2 |
| Salmonella bacteraemia – non-typhi | 1 | 0 | 1 |
| Secondary/tertiary syphilis | 1 | 0 | 1 |
| Severe malnutrition | 0 | 1 | 1 |
| Stevens-Johnson Syndrome | 2 | 3 | 3 |
| Stroke, cerebrovascular accident | 3 | 5 | 6 |
| Thrombocytopenia | 0 | 18 | 18 |
| Trauma | 1 | 1 | 1 |
| Ulcer, decubitus ulcer | 0 | 1 | 1 |
| Vaginal bleeding | 0 | 1 | 1 |
| Vomiting | 0 | 5 | 5 |
| * primary or secondary cause of death  ** patients could have more than one event on the same day, in which case all are included | | | |

**Supplementary Table 3 Baseline predictors of deaths from cryptococcosis and unascertained causes**

|  | **Deaths from cryptococcosis** | | **Deaths from unascertained causes** | |
| --- | --- | --- | --- | --- |
|  | **sHR [95% CI]** | **p** | **sHR [95% CI]** | **p** |
| Enhanced-prophylaxis vs standard-prophylaxis | 0.34 [0.12-0.92] | 0.03 | 0.59 [0.37-0.96] | 0.03 |
| CD4 (per 10 cell/mm^3^ higher) | 0.77 [0.62-0.95] | 0.02 | 0.91 [0.83-1.00] | 0.05 |
| Creatinine clearance (per 10 ml/min higher) |  | - |  | <0.001 |
| <75 ml/min | - | - | 0.70 [0.59-0.83] | <0.001 |
| ≥75 ml/min | - | - | 1.03 [0.97-1.10] | 0.29 |
| Bilirubin (per umol/l higher) | - | - |  | 0.05 |
| <5 umol/l | - | - | 1.21 [0.97-1.49] | 0.08 |
| ≥5 umol/l | - | - | 1.02 [0.99-1.06] | 0.15 |
| Albumin (per g/l higher) | - | - | 0.95 [0.91-0.99] | 0.01 |
| Previous healthcare contact | - | - | 0.27 [0.08-0.92] | 0.04 |
| Current wasting/severe weight loss (WHO 3/4) | - | - | 2.00 [1.14-3.50] | 0.02 |
| Participant-reported fever, yes vs no | - | - | 1.63 [0.95-2.81] | 0.08 |
| Participant-reported vomiting, yes vs no | 4.15 [1.53-11.31] | 0.005 | - | - |
| EQ-5D mobility, vs. no problems |  | - |  | 0.05 |
| Some problems | - | - | 1.75 [0.96-3.20] | 0.07 |
| Confined to bed | - | - | 5.79 [1.25-26.70] | 0.02 |
| EQ-5D self-care, vs. no problems |  | - |  | 0.05 |
| Some problems | - | - | 2.24 [1.13-4.45] | 0.02 |
| Unable to wash/dress | - | - | 1.46 [0.34-6.16] | 0.61 |
| Backwards elimination with exit p=0.1 including non-linearity based on fractional polynomials where p<0.05 (Stata mfp), forcing randomized group into the models. Other factors considered were those in Table 1 or identified as predictors of death from any cause in the companion paper in this supplement[5]. Final models re-fit to all observations with complete data for chosen factors  sHR=subhazard ratio. Adjusted for other factors in column; model for deaths from unascertained causes also adjusted for centre (p=0.03) | | | | |

**Supplementary Table 4 Last post-baseline CD4 and viral load before fatal and non-fatal events of each cause**

|  | **CD4** | | | **Viral load** | | |
| --- | --- | --- | --- | --- | --- | --- |
|  | **Number in follow-up at week 4*** | **Number with post-baseline measurement** | **Median (IQR)** | **Number in follow-up at week 4*** | **Number with post-baseline measurement** | **Median (IQR)** |
| Deaths |  |  |  |  |  |  |
| Tuberculosis | 54 | 45 | 53 (32-108) | 55 | 46 | 125 (<50-314) |
| Cryptococcosis | 15 | 14 | 46 (14-59) | 15 | 14 | <50 (<50-212) |
| Severe bacterial infection | 19 | 19 | 53 (14-91) | 20 | 18 | 70 (<50-522) |
| Potentially-azithromycin-responsive infection | 13 | 11 | 50 (32-115) | 15 | 13 | 309 (118-637) |
| Other event | 47 | 44 | 66 (36-111) | 49 | 45 | 99 (<50-322) |
| Unknown cause | 65 | 55 | 67 (32-99) | 65 | 54 | 70 (<50-724) |
| All | 158 | 137 | 59 (32-101) | 161 | 140 | 95 (<50-522) |
| Non-fatal events |  |  |  |  |  |  |
| Tuberculosis | 79 | 73 | 67 (41-116) | 80 | 73 | 110 (<50-530) |
| Cryptococcosis | 20 | 20 | 78 (14-109) | 21 | 20 | <50 (<50-108) |
| Severe bacterial infection | 44 | 43 | 65 (25-98) | 45 | 44 | 134 (<50-3874) |
| Potentially-azithromycin-responsive infection | 38 | 36 | 60 (36-112) | 38 | 36 | 681 (<50-33213) |
| Other event | 367 | 359 | 84 (45-143) | 368 | 361 | 113 (<50-690) |

*Or alive and in follow-up post-week 2 and with post-baseline measurement. First post-baseline VL and CD4 measured at week 4, so patients dying or lost to follow-up before this timepoint cannot contribute to this analysis.

Note: categories are not mutually exclusive. For example, a death adjudicated by the Endpoint Review Committee as due to cryptococcal meningitis and cirrhosis would be classified as death due to cryptococcosis and to ‘other’ events. Consequently statistical significance testing cannot be conducted to compare categories

**Supplementary Table 5 Details of final models**

|  | **Number of interior knots for baseline hazard^a^** | **Effect of enhanced-prophylaxis versus standard-prophylaxis** |
| --- | --- | --- |
| Deaths |  |  |
| Tuberculosis | 3 | Non-proportional hazards (2 interior knots) |
| Cryptococcosis | 1 | Proportional hazards |
| Severe bacterial infection | 2 | Proportional hazards |
| Potentially-azithromycin-responsive infection | 2 | Non-proportional hazards (linear effect of log time) |
| Other event | 1 | Proportional hazards |
| Unknown cause | 1 | Proportional hazards |
| IRIS-compatible | 2 | Proportional hazards |
| All events |  |  |
| Tuberculosis | 3 | Proportional hazards |
| Cryptococcosis | 2 | Proportional hazards |
| Severe bacterial infection | 3 | Proportional hazards |
| Potentially-azithromycin-responsive infection | 2 | Proportional hazards |
| Other event | 4 | Proportional hazards |
| IRIS-compatible | 3 | Proportional hazards |
| ^a^ One interior knot at the 50th percentile of the uncensored survival times, two at the 33rd and 67th, three at the 25th, 50th and 75th or four at the 20th, 40th, 60th and 80th; plus two boundary knots at their minimum and maximum. | | |

**Supplementary Figure 1 Cumulative incidence of causes of death**

sHR=subhazard ratio (unadjusted)

Note: categories are not mutually exclusive. For example, a death adjudicated by the Endpoint Review Committee as due to cryptococcal meningitis and cirrhosis would be classified as death due to cryptococcosis and to ‘other’ events

Supplementary Figure 2 Cumulative incidence of first fatal or non-fatal (grade 3/4 AE, SAE, WHO 3/4) event

sHR=subhazard ratio (unadjusted)

Note: categories are not mutually exclusive. For example, an SAE adjudicated by the Endpoint Review Committee as due to cryptococcal meningitis and cirrhosis would be classified as cryptococcosis and an ‘other’ event. All-cause mortality shown in Figure 2(b) of [4].

SUPPLEMENTARY REFERENCES

1. Fine JP, Gray RJ. A Proportional Hazards Model for the Subdistribution of a Competing Risk. Journal of the American Statistical Association **1999**; 94(446): 496-509.

2. Royston P, Parmar MKB. Flexible parametric proportional-hazards and proportional-odds models for censored survival data, with application to prognostic modelling and estimation of treatment effects. Statistics in Medicine **2002**; 21: 2175-97.

3. Lambert PC, Royston P. Further development of flexible parametric models for survival analysis. The Stata Journal **2009**; 9(2): 265-90.

4. Hakim J, Musiime V, Szubert A, et al. Enhanced Prophylaxis plus Antiretroviral Therapy for Advanced HIV Infection in Africa. New England Journal of Medicine **2017**; 377(3): 233-45.

5. Siika A, McCabe L, Bwakura-Dangarembizi M, et al. LATE PRESENTATION WITH HIV IN AFRICA: PHENOTYPES, RISK AND RISK STRATIFICATION IN THE REALITY TRIAL. Clinical Infectious Diseases.
